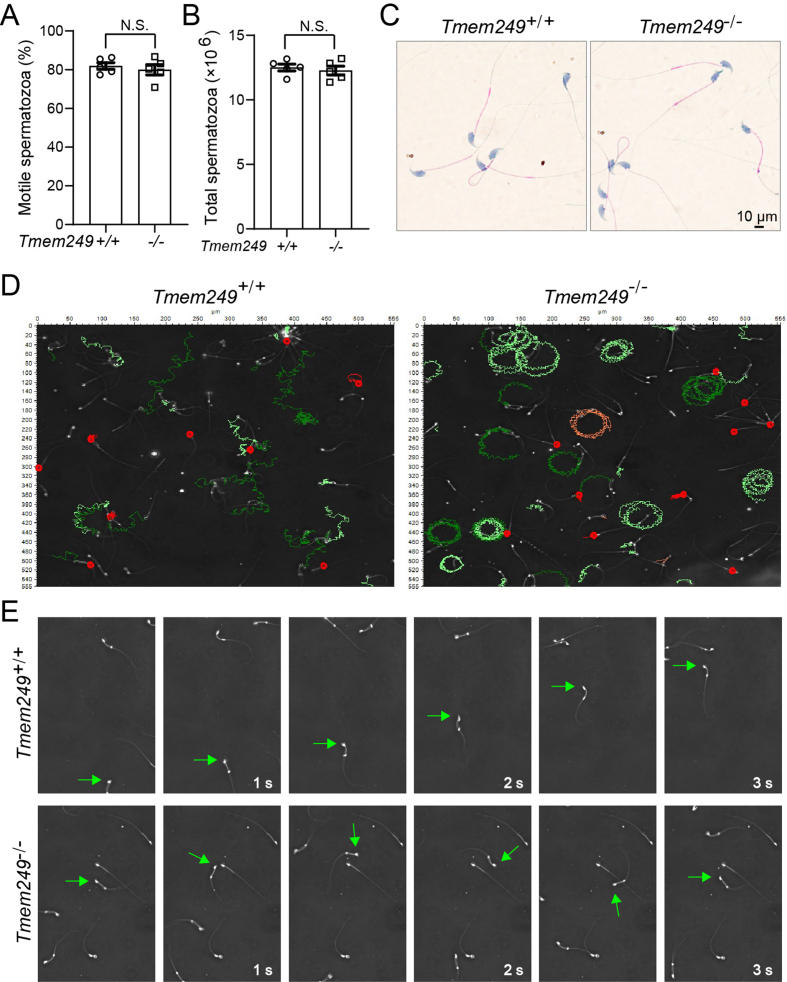# TMEM249-mediated sperm hyperactivation is required for mouse fertility

**DOI:** 10.1016/j.gendis.2025.101559

**Published:** 2025-02-18

**Authors:** Xiuge Wang, Xiaochao Wei, Yaping Gao, Chunhong Yang, Qiang Jiang, Yao Xiao, Jinpeng Wang, Yaran Zhang, Zhihua Ju, Jinming Huang

**Affiliations:** a Key Laboratory of Livestock and Poultry Multi-omics of MARA, Institute of Animal Science and Veterinary Medicine, Shandong Academy of Agricultural Sciences, Jinan, Shandong 250100, China; b Technical Innovation Center of Dairy Cattle Breeding Industry of Shandong Province, Institute of Animal Science and Veterinary Medicine, Shandong Academy of Agricultural Sciences, Jinan, Shandong 250100, China; c Shandong Key Laboratory of Animal Disease Control and Breeding, Institute of Animal Science and Veterinary Medicine, Shandong Academy of Agricultural Sciences, Jinan, Shandong 250100, China

Fertilization is a fundamental biological phenomenon essential for the initiation of new life. This process encompasses sperm hyperactivation, acrosome reaction, and sperm–egg fusion, all of which are intricately controlled by Ca^2+^ signaling.^1^ The cation channel CatSper, predominantly situated in the flagellar region of mature sperm, plays a pivotal role in mediating various Ca^2+^-dependent physiological events crucial for sperm activation and fertility.^2^ In animals, the motility of sperm plays a vital role in determining reproductive efficiency and overall productivity. Sperm must undergo changes in the female to fertilize eggs, including hyperactivated motility in the tail, triggered by calcium ions entering through CatSper protein.^3^ In hyperactivation, sperm tail movement shifts from fast and symmetrical to slow and asymmetrical. Recent findings suggest that the transmembrane protein 249 (TMEM249) may serve as an additional component of CatSper.^4^

We first predicted that the amino acid sequence of TMEM249 contained five conserved domains, thus locating at three distinct regions (extracellular, transmembrane, and cytoplasmic) on the sperm membrane (Figs. S1A–C). Additionally, through comparative analysis of TMEM249 amino acid sequences across various mammalian species, a high degree of conservation was observed (Fig. S1D). To assess the potential role of TMEM249 in male fertility, the expressed level of *Tmem2*49 mRNA was initially examined in various tissues of mice, revealing specific expression in the testis as determined by reverse transcription PCR analysis (Fig. 1A). The expression of *Tmem249* was observed to commence at postnatal day 18 in mice, coinciding with the appearance of round spermatids in the seminiferous tubules (Fig. 1B). These results suggest that TMEM249 on sperm membrane may be involved in the process of mouse spermatogenesis.

**Figure 1:**
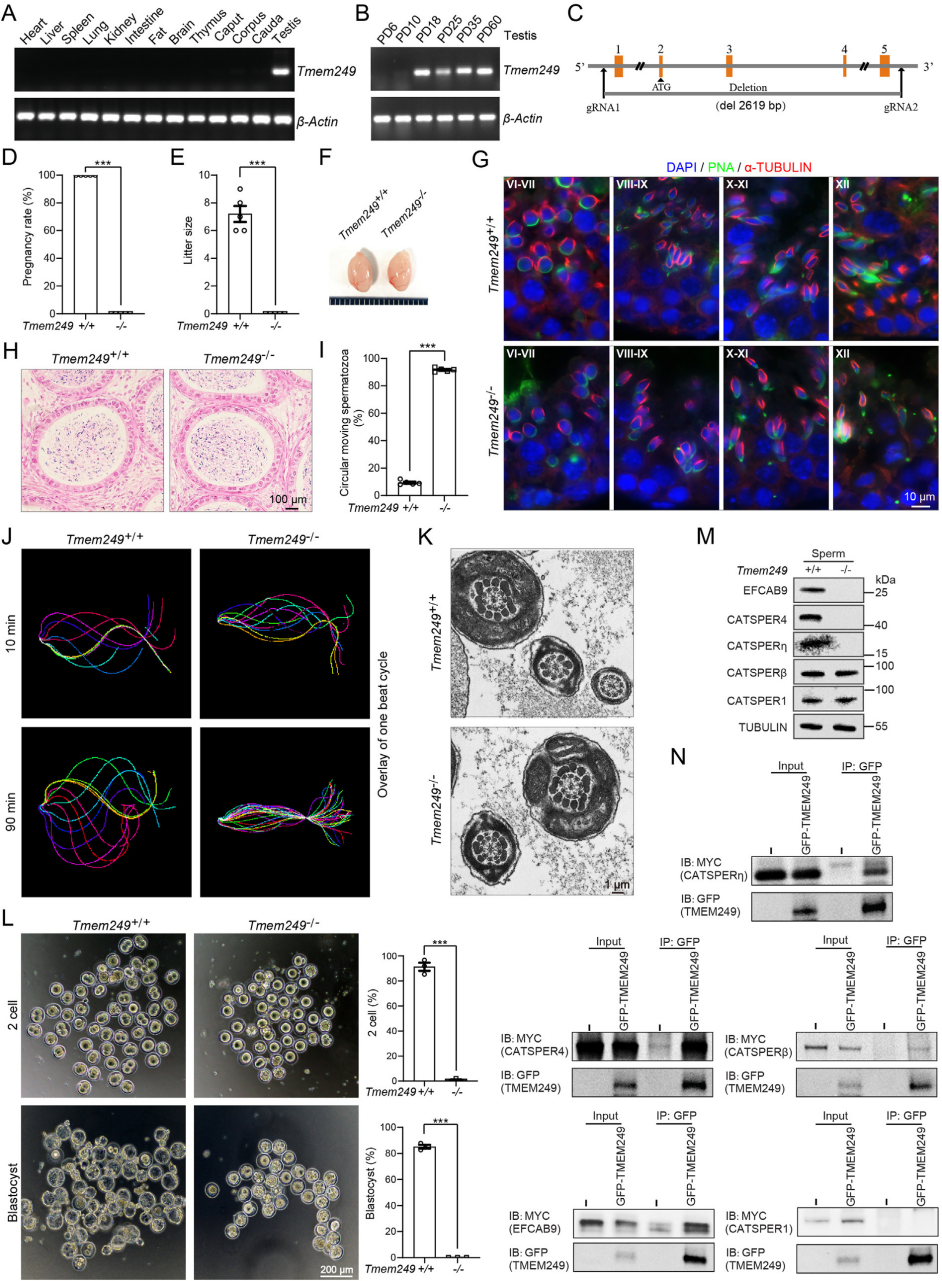
TMEM249-mediated sperm hyperactivation is required for mouse fertility. **(A)** The expression level of *Tmem2*49 mRNA in different mouse tissues. The *β-Actin* was used as the control. **(B)** The expression level of *Tmem2*49 mRNA at postnatal day 6, 10, 18, 25, 35, and 60 in mouse testes. The *β-Actin* was used as the control. **(C)** Construction strategy of *Tmem249-*knockout mouse. **(D)** The conception rates of male mice with *Tmem249*^*+/+*^ and *Tmem249*^*−*/−^ genotypes when mated with wild female mice. **(E)** The litter size of female mice mated with male mice of different *Tmem249* genotypes. **(F)** Testicular size of mice before and after the knockout of *Tmem249*. **(G)** Immunofluorescence staining on seminiferous tubules at different developmental stages in *Tmem249*^*+/+*^ and *Tmem249*^*−*/−^ mice. α-TUBULIN stained the spermatid manchette structure (in red), PNA staining highlighted the acrosome (in green), and DAPI staining was utilized to identify the nucleus (in blue). The different stages of seminiferous tubules are indicated by Roman numerals. **(H)** Hematoxylin–eosin staining of cauda epididymis from *Tmem249*^*+/+*^and *Tmem249*^*−*/−^ mice. The results revealed no significant abnormalities in the spermatozoa of the cauda epididymis in *Tmem249-*knockout mice. **(I)** The percentage of abnormal circular motion in motile sperm from *Tmem249*^*+/+*^ and *Tmem249*^*−*/−^ mice. **(J)** Flagellar waveform of *Tmem249*^*+/+*^ and *Tmem249*^−/−^ sperm cells. Videos were captured at 200 frames per second, depicting head-tethered cells interacting with glass coverslips incubating under capacitated conditions at time points of 10 min and 90 min. The flagellar traces from one beat cycle were overlaid and color-coded to represent time progression. **(K)** Transmission electron microscopy was used to examine the internal flagellar structures of epididymal spermatozoa from *Tmem249*^*+/+*^ and *Tmem249*^*−*/−^ mice. **(L)** Representative images of 2-cell embryos and blastocysts. Oocytes were co-incubated with sperm from *Tmem249*^*+/+*^ and *Tmem249*^*−*/−^ mice for 6 h. The rates of the 2-cell embryo and blastocyst formation following *in vitro* fertilization were counted. **(M)** Expression levels of CatSper subunits in *Tmem249*^*−*/−^ sperm cells compared with those of *Tmem249*^*+/+*^ sperm cells. **(N)** TMEM249-GFP was co-transfected with EFCAB9-MYC, CATSPER4-MYC, CATSPERη-MYC, CATSPERβ-MYC, or CATSPER1-MYC into HEK293T cells, followed by immunoblotting of the co-immunoprecipitation complexes using anti-GFP and anti-MYC antibodies.

To investigate the impact of TMEM249 on male fertility, we targeted and deleted a 2619 bp sequence encompassing the 1–5 exons of *Tmem249* to construct the knockout mice utilizing CRISPR/Cas9 technology (Fig. 1C). Subsequent western blotting and reverse transcription PCR analyses were conducted to confirm the complete disruption of *Tmem249* expression in *Tmem249*-deficient mice (Figs. S2A–C). Despite the ability of *Tmem249*-knockout males to successfully mate with wild-type females, none of the females achieved pregnancy (Fig. 1D and E). Additionally, male mice deficient in *Tmem249* did not exhibit notable variations in individual morphology, body weight, and testis dimensions in comparison to *Tmem249*^*+/+*^ counterparts (Fig. 1F; Figs. S2A–D). These findings indicate the essential role of *Tmem249* in male fertility and suggest its potential involvement in spermatogenesis.

The objective of our study is to investigate the impact of disrupting *Tmem249* on male fertility. However, our findings indicated that there were no alterations in the histological composition of testis sections in *Tmem249*^*−*/−^ mice as revealed by hematoxylin–eosin staining (Fig. S3E). Additionally, normal germ cell types, morphology, and stages of the epithelial cycle in the seminiferous tubules were observed in *Tmem249*^*−*/−^ mouse testis using periodic acid-Schiff and hematoxylin-stained sections (Fig. S3F). Examination of the morphology of the 16 steps of spermatogenesis in the testis using PNA and TUBULIN did not indicate any abnormalities (Fig. 1G). Furthermore, the analysis of meiotic prophase I progression in spermatocytes using SYCP3 and γH2AX markers did not reveal any discernible differences between the two genotypes (Fig. S3G). Therefore, it can be inferred that male infertility resulting from *Tmem249* deficiency is not attributed to spermatid defects occurring during spermatogenesis.

Furthermore, the research indicated that there were no significant alterations in spermatozoa morphology and quantity in the cauda epididymis of *Tmem249*-knockout male mice when compared with control mice (Fig. 1H). The morphology, motility, and total count of spermatozoa from the unilateral cauda epididymis in *Tmem249*^*−*/−^ mice were also normal (Figs. S4A–C). Although there was no statistically significant alteration in sperm motility observed in *Tmem249*-knockout mice, approximately 90% of motile sperm exhibited circular motion (Fig. 1I; Fig. S4D, E). Our study further revealed that sperm lacking *Tmem249* displayed stiffness in the midpiece of the flagellum following 10 or 90 min of exposure to capacitated conditions (Video S1, 2).

Supplementary video related to this article can be found at https://doi.org/10.1016/j.gendis.2025.101559

The following are the supplementary data related to this article:

VideoS1 2 VideoS1

VideoS2 VideoS2

VideoS3 VideoS3

VideoS4 VideoS4

VideoS5 VideoS5

VideoS6 VideoS6

Additionally, *Tmem249*-deficient sperm exhibited inflexible flagella that could only beat at the distal end of the tail by the experiment of tethering the sperm head to the culture dish bottom (Fig. 1J; Videos S3–6). The internal organization of sperm flagella, particularly the configuration of “9 + 2” microtubules, is crucial for determining sperm motility. However, subsequent analysis of the flagellum ultrastructure in the knockout mice using transmission electron microscopy revealed no apparent abnormalities in the sperm flagellum structure (Fig. 1K). To address the fertilization potential of epididymal *Tmem249*^*−*/−^ sperm, an *in vitro* fertilization analysis was performed to assess the ratio of two-cell and blastocyst embryos to total oocytes. The findings demonstrated a significant decrease in the fertilization capacity of *Tmem249*^*−*/−^ sperm, suggesting that TMEM249 plays a crucial role in mediating the interaction between sperm and egg during fertilization (Fig. 1L).

CatSper contains the most subunits of any known ion channel, including CATSPER1–4 and auxiliary proteins like CATSPERβ, γ, δ, ε, ζ, and EFCAB9.^5^ Consequently, our initial inquiry involved the assessment of the presence of the CatSper channel in TMEM249-deletion spermatozoa through the examination of protein levels of CatSper subunits. Immunoblot analyses indicate the absence of the previously identified CatSper components (EFCAB9, CATSPER4, CATSPERη, CATSPERβ) in TMEM249-deletion spermatozoa (Fig. 1M), consistent with findings in other CatSper subunit-null spermatozoa.^3^^,^^5^ To test the impact of TMEM249 deficiency on the presence of CatSper components, we conducted an over-expression experiment of these proteins in HEK 293T cells to assess potential interactions through co-immunoprecipitation. Our findings indicate that TMEM249 can directly interact with these CatSper components that are typically absent (Fig. 1N). Moreover, our experimental findings indicate that the deletion of TMEM249 does not substantially alter the expression levels of CATSPER1 and CATSPERβ (Fig. 1M), which contradicts earlier studies.^4^ We hypothesize that this discrepancy may arise from the lack of direct interaction between TMEM249 and CATSPER1, and while CATSPERβ exhibits a relatively weak binding affinity, it is insufficient to influence the expression of CATSPERβ. These results imply that the deletion of TMEM249 could potentially disrupt the stability of the cation channel CatSper within sperm flagella.

TMEM249 has been identified as a novel component of the CatSper complex, and its deletion in mice leads to infertility. This study aims to elucidate the potential role of TMEM249 by generating a *Tmem249*-knockout mouse model through the CRISPR/Cas9 system. Our findings demonstrate that male mice lacking *Tmem249* are infertile, with spermatozoa exhibiting stiff flagella along the midpiece to the proximal region of the principal piece, potentially contributing to the observed infertility. The lack of mouse TMEM249 resulted in the destabilization of interacting proteins associated with the CatSper components in the sperm flagella. Our research indicates that TMEM249 likely contributes to sperm hyperactivation by maintaining the stability of the cation channel CatSper within the sperm flagella. The results of these experiments contribute to our knowledge of the specific role of TMEM249 ion channel protein in male fertility and help uncover the underlying mechanisms involved. This allows researchers to establish a connection between *Tmem249* variants and male infertility, providing clinical relevance and potential diagnostic implications.

## CRediT authorship contribution statement

**Xiuge Wang:** Writing – review & editing, Writing – original draft, Funding acquisition. **Xiaochao Wei:** Writing – original draft, Data curation. **Yaping Gao:** Investigation, Data curation. **Chunhong Yang:** Software, Investigation. **Qiang Jiang:** Validation, Software. **Yao Xiao:** Investigation, Formal analysis. **Jinpeng Wang:** Software. **Yaran Zhang:** Software, Resources. **Zhihua Ju:** Visualization, Supervision. **Jinming Huang:** Writing – review & editing, Writing – original draft.

## Ethics declaration

The experimental procedures involving animals were approved (approved number: IIASVM-2024-043) by the Animal Experimental Ethics Committee, Institute of Animal Science and Veterinary Medicine, Shandong Academy of Agricultural Sciences.

## Funding

This study was supported by the 10.13039/501100001809National Natural Science Foundation of China (No. 32272854, 32202677), Biological Breeding-Major Projects in National Science and Technology (China) (No. 2023ZD04049), 10.13039/501100012166National Key Research and Development Program of China (No. 2021YFF1000700), Shandong Agricultural Elite Variety Project (China) (No. 2022LZGC012), and Agricultural Scientific and Technological Innovation Project of Shandong Academy of Agricultural Sciences (China) (No. CXGC2024A03, CXGC2024B03).

## Conflict of interests

The authors declared no competing financial and non-financial interests.

## Figures and Tables

**figs1:**
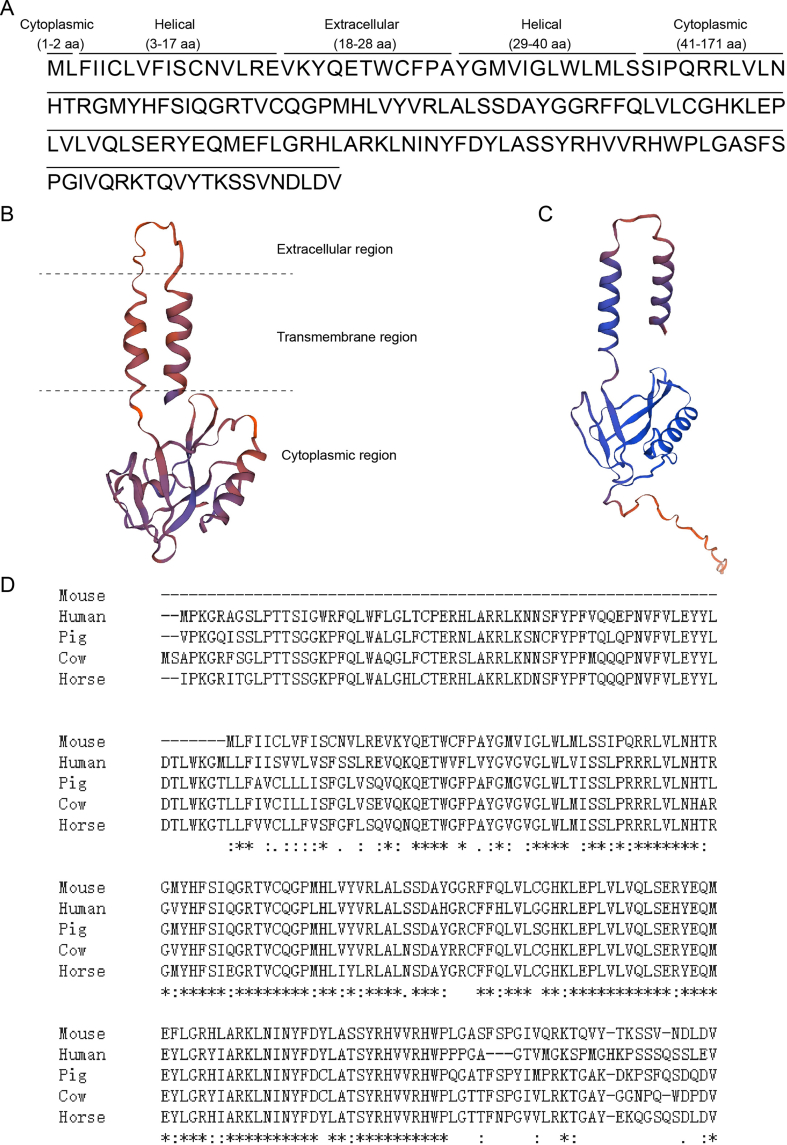


**figs2:**
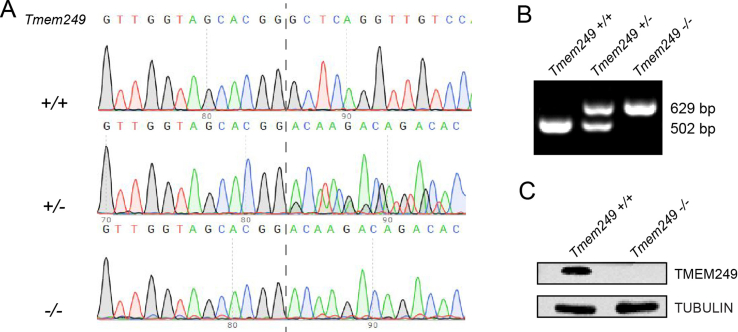


**figs3:**
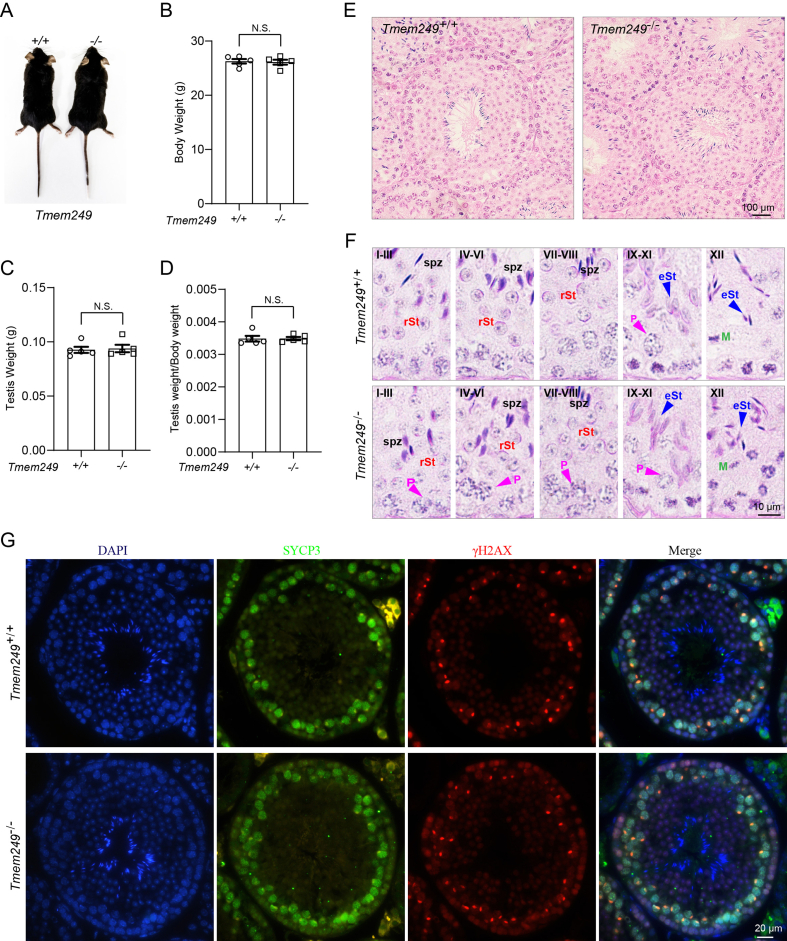


**figs4:**