# Supplementary material for: TMEM249-mediated sperm hyperactivation is required for mouse fertility
Source: Genes Dis. 2025 Feb 18;13(1):101559. doi: 10.1016/j.gendis.2025.101559 (PMC12624607; doi:10.1016/j.gendis.2025.101559)
Supplement: Multimedia component 1 [file mmc1.doc]

**Supplementary Data**

**Materials and Methods**

**Animals**

The *Tmem249*-knockout mouse model was created utilizing CRISPR/Cas9 technology. The PCR primers employed for genotyping mice were as follows: F1, 5’-TGTATGGTCCTTAGAGTAAGTGGTTG-3’; R1, 5’-CCAGAAATTGTGGGCGT

ATTCCT-3’; R2, 5’-CCCACTGTCTTTATACAGGGCTCA-3’. The testicular tissue was preserved in Bouin’s solution and 4% paraformaldehyde (PFA) for subsequent analysis. A portion of mouse testis was quickly sectioned into small portions and stored by freezing in liquid nitrogen. The experimental procedures involving animals were approved (approved number: IIASVM-2024-043) by the Animal Experimental Ethics Committee, Institute of Animal Science and Veterinary Medicine, Shandong Academy of Agricultural Sciences.

**RNA extraction and RT-PCR**

Mouse testis RNA was extracted using a RNA Extraction Kit (TIANGEN, 4992858) and cDNA was synthesized with the PrimeScript RT Reagent kit (Takara, RR037A). The RT-PCR primer sets used were as follows: *Tmem249* primers (5’-TAGAGCCACTTGTGCTGGTG-3’ and 5’-GTGGCGGACTACATGACGAT-3’) and *β-Actin* primers (5’-GGCTGTATTCCCCTCCATCG-3’ and 5’-CCAGTTGGTAA

CAATGCCATGT-3’). The RT-PCR reactions were carried out at 94°C for 5 min, followed by 28 cycles at 94°C for 30 s, 60°C for 30 s, and 72°C for 20 s, with a final extension at 72°C for 10 min.

**Analysis of sperm quality**

The unilateral cauda epididymis of mice was promptly dissected into fragments in 1 ml pre-warmed TYH medium (Aibei, M2030) and incubated for 10 minutes at 37°C (5% CO_2_). Sperm motility parameters were assessed using a computer-assisted sperm analyzer (Minitube, AndroVision), while sperm count was determined using a hemocytometer under an Axio Scope A1 light microscope (Zeiss) with another unilateral cauda epididymis of mice.

**Immunofluorescence (IF)**

The IF experiment follows our previous procedure^1^. Simply, fixed PFA tissues were sectioned, washed with PBS, treated with Triton-X 100, washed again with PBS, blocked with 5% BSA, and finally treated with primary antibody. The primary antibody was washed and then incubated with the secondary antibody at 37°C for 1 hour. After washing with PBS, the nuclei was stained with DAPI (Beyotime, C1005) for 5 minutes, followed by washing to prepare sample imaging. Finally, the sample was ready for observation and image acquisition.

**Antibodies**

The primary antibodies utilized in this study for immunoblotting, immunofluorescence, or co-immunoprecipitation were as follows: anti-TMEM249, anti-EFCAB9, anti-CATSPER4 and anti-CATSPERη antibodies were generated by Dia-an Biotech (Wuhan, China); anti-α-TUBULIN (ABclonal, AC012); PNA (Thermo, L21409); anti-SYCP3 (Abcam, ab97672); anti-γH2A.X (HUABio, ET1602-2); anti-CATSPERβ (Abmart, TD9349S); anti-CATSPER1 (Abmart, TD9352S); anti-Tag-GFP (Abmart, M20004S); anti-Tag-MYC (Proteintech, 16286-1-AP); anti-mouse Alexa Fluor 488 (Thermo, A32766); anti-rabbit Alexa Fluor 555 (Thermo, A32794); anti-mouse-HRP (ZSGB-BIO, zb-2305); anti-rabbit-HRP (Abcam, ab6721); anti-rabbit-IRDye 800CW (LI-COR, 827-08365); anti-mouse-IRDye 680RD (LI-COR, 926-68170).

**Transmission electron microscopy (TEM)**

The sperm samples obtained from adult mice were processed for the TEM analysis by fixation in a buffer solution with a pH of 7.2 containing 1.5% glutaraldehyde, 1.5% paraformaldehyde (PFA), and 0.1 M cacodylic acid sodium salt trihydrate. Subsequent to fixation, the samples underwent treatment with 1% OsO4 for 1 hour, dehydration in a series of acetone solutions, and embedding in resin. Ultrathin sections were then prepared using an ultramicrotome (Leica, EM UC7) and subsequently examined and imaged with a transmission electron microscope (Hitachi, HT7800).

**Flagellar waveform of sperm cells**

To induce hyperactivation, sperm from the epididymis were incubated in TYH medium for 10 or 90 minutes at 37˚C (5% CO_2_). These hyperactivated sperm were placed on a fibronectin-coated dish in a controlled temperature chamber for 10-20 minutes. Then, the sperm head is tethered to the dish bottom, only letting the flagella free swing. Their flagellar movements were recorded for more than 2 seconds at 200 frames per second using a high-speed camera attached to an AndroVision microscope (Minitube). SpermQ software^2^ was used to measure beating frequency and create images to track sperm flagella movement.

**Co-immunoprecipitation (Co-IP)**

The coding regions of CatSper subunits were individually cloned into either the pCMV-MYC or pcDNA3.1-GFP vector, followed by transient co-transfection of the expression vectors into HEK 293T cells using Lipofectamine 3000. After 36-48 hours post-transfection, cells were harvested and lysed in TAP buffer containing 10% glycerol, 50 mM HEPES, 100 mM KCl, 2 mM EDTA, 0.1% NP-40, 10 mM NaF, 0.25 mM Na3VO4, 50 mM β-glycerolphosphate, 2 mM DTT, 1× protease inhibitor cocktail, at pH 7.4. Subsequently, cell lysates were subjected to incubation with anti-GFP antibody and rotation overnight at 4℃. Following this, 30 µl of protein A-Sepharose (GE, 17-1279-03) was introduced to each incubation sample for a duration of 2-3 hours at 4℃. The resulting pellet was then washed with TAP buffer, and the co-immunoprecipitated proteins were eluted using 2×SDS loading buffer for 10 minutes at 95℃ before being further analyzed through immunoblotting.

**Statistical analysis**

Statistical analyses of mouse phenotypic data were conducted using GraphPad Prism version 8.2.1 and an unpaired two-tailed Student’s t test. Error bars represent the mean ± SEM. Statistical significance was determined based on *P* values less than 0.001 (***). N.S. means no significant difference. Each experiment was replicated a minimum of three times.

**References**

1. Wei X, Wang X, Yang C, et al. CFAP58 is involved in the sperm head shaping and flagellogenesis of cattle and mice. Development. 2024;151(7):dev202608.
2. Hansen JN, Rassmann S, Jikeli JF, Wachten D. SpermQ-A Simple Analysis Software to Comprehensively Study Flagellar Beating and Sperm Steering. Cells. 2018;8(1):10.
3. Jumper J, Evans R, Pritzel A, et al. Highly accurate protein structure prediction with AlphaFold. Nature. 2021;596(7873):583-589.
4. Madeira F, Madhusoodanan N, Lee J, et al. The EMBL-EBI Job Dispatcher sequence analysis tools framework in 2024. Nucleic Acids Res. 2024;52(W1):W521-W525.

**
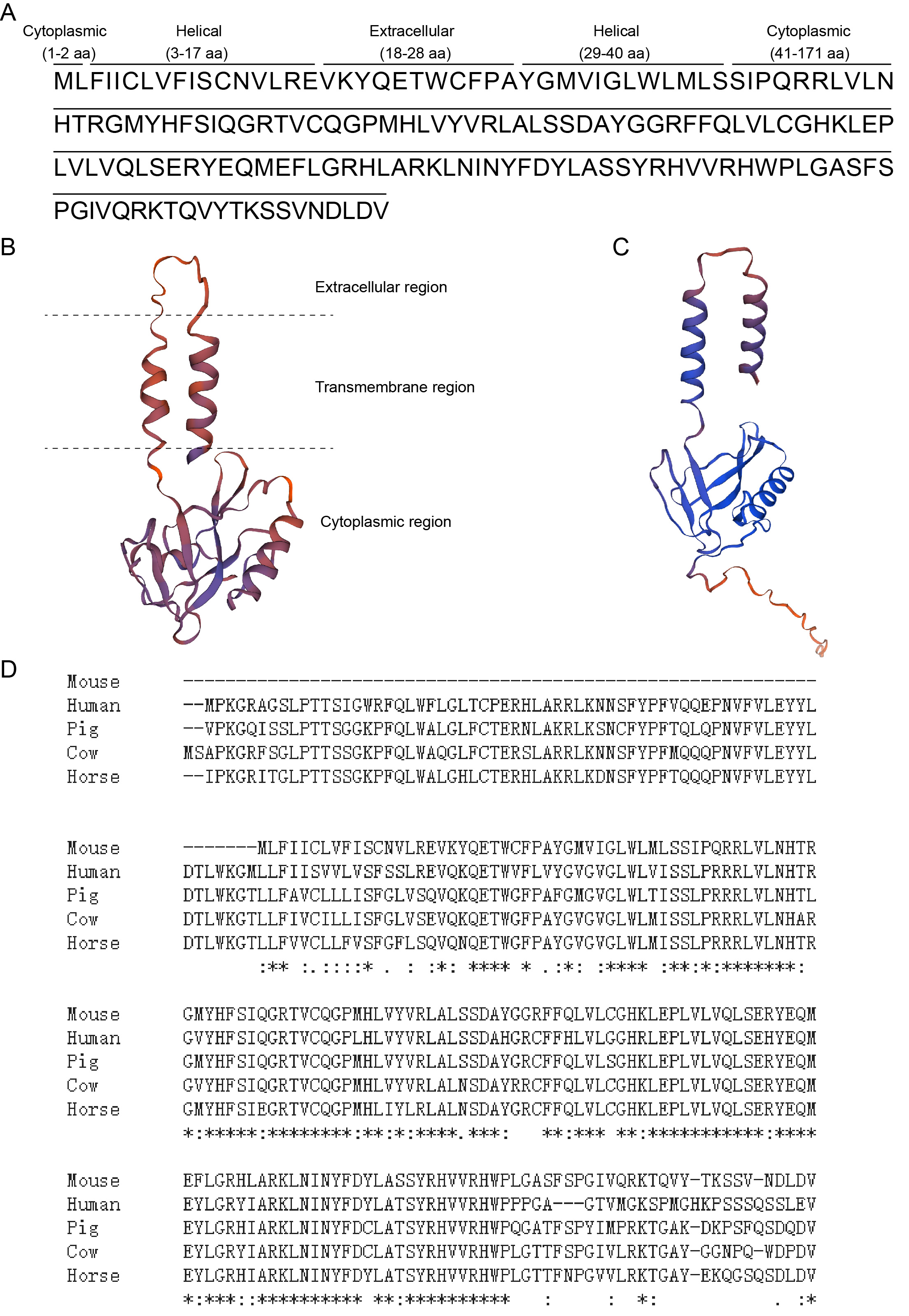
Fig. S1**. The analysis of TMEM249 amino acid sequence. (A) The predicted domain structure of mouse TMEM249 protein. Mouse TMEM249 (Ensembl: ENSMUSP00000155040.2) contains two cytoplasmic, two helical and one extracellular domains by the analysis of the UniProtKB database. (B) TMEM249 domains constituted three distinct regions predicted by Swiss-Model based on 7eeb.1. (C) TMEM249 structure (AF-A0A2R8VHF7-F1-model-v2) was predicted by AlphaFold^3^. (D) Multiple sequence alignment of the mammal TMEM249 by Clustal Omega^4^. The amino acids of TMEM249 from mouse, human, pig, cow and horse are highly conserved.


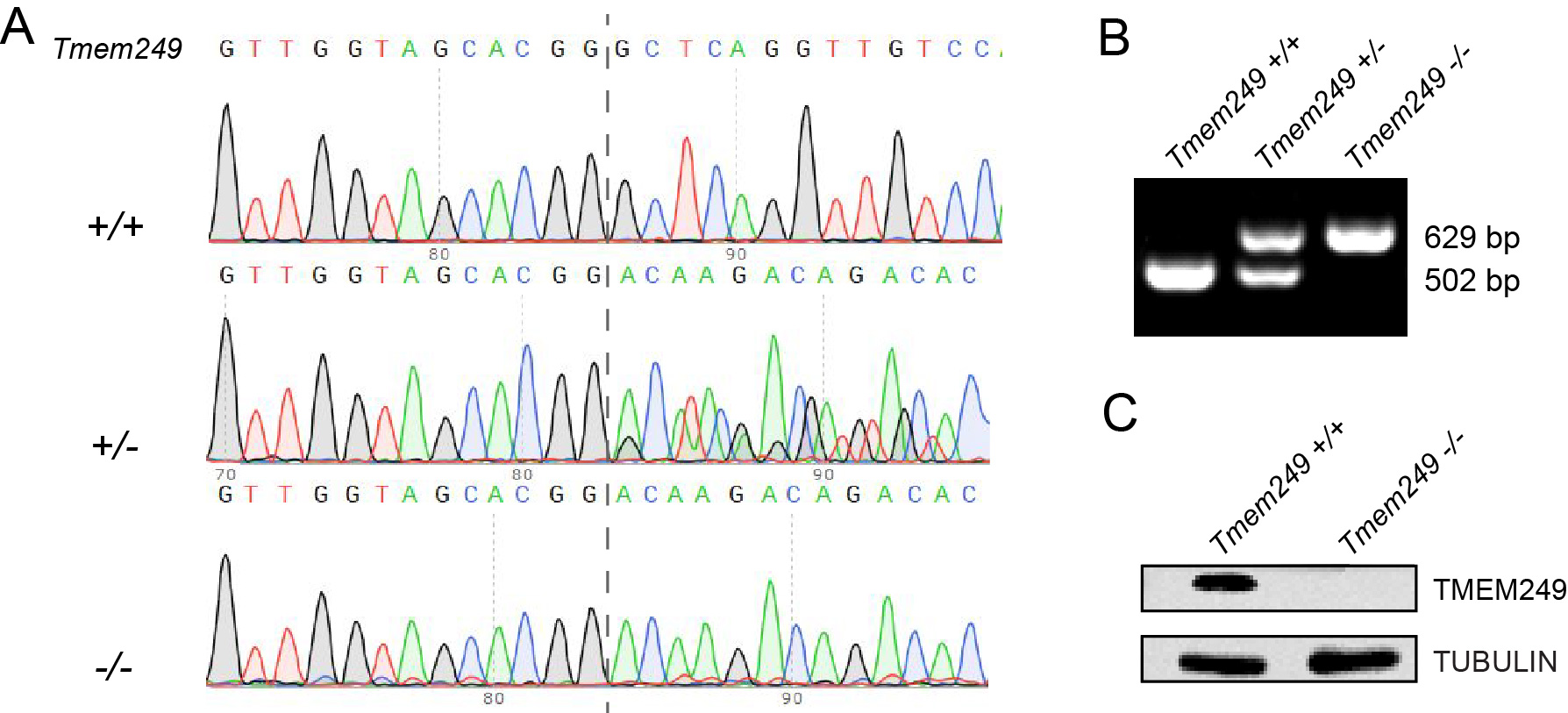


**Fig. S2.** Generation of *Tmem249*-knockout mice. (A) Sequencing chromatographs of PCR products amplifying the *Tmem249* sequence of different genotype mice. (B) Genotyping identification of *Tmem249* mice. The *Tmem249*^+/+^ generated a 502 bp band, and the *Tmem249*^+/-^ produced the 629 bp and 502 bp bands, and the *Tmem249*^-/-^ produced a 629 bp band. (C) Immunoblotting of TMEM249 in testes. TUBULIN was used as the control.


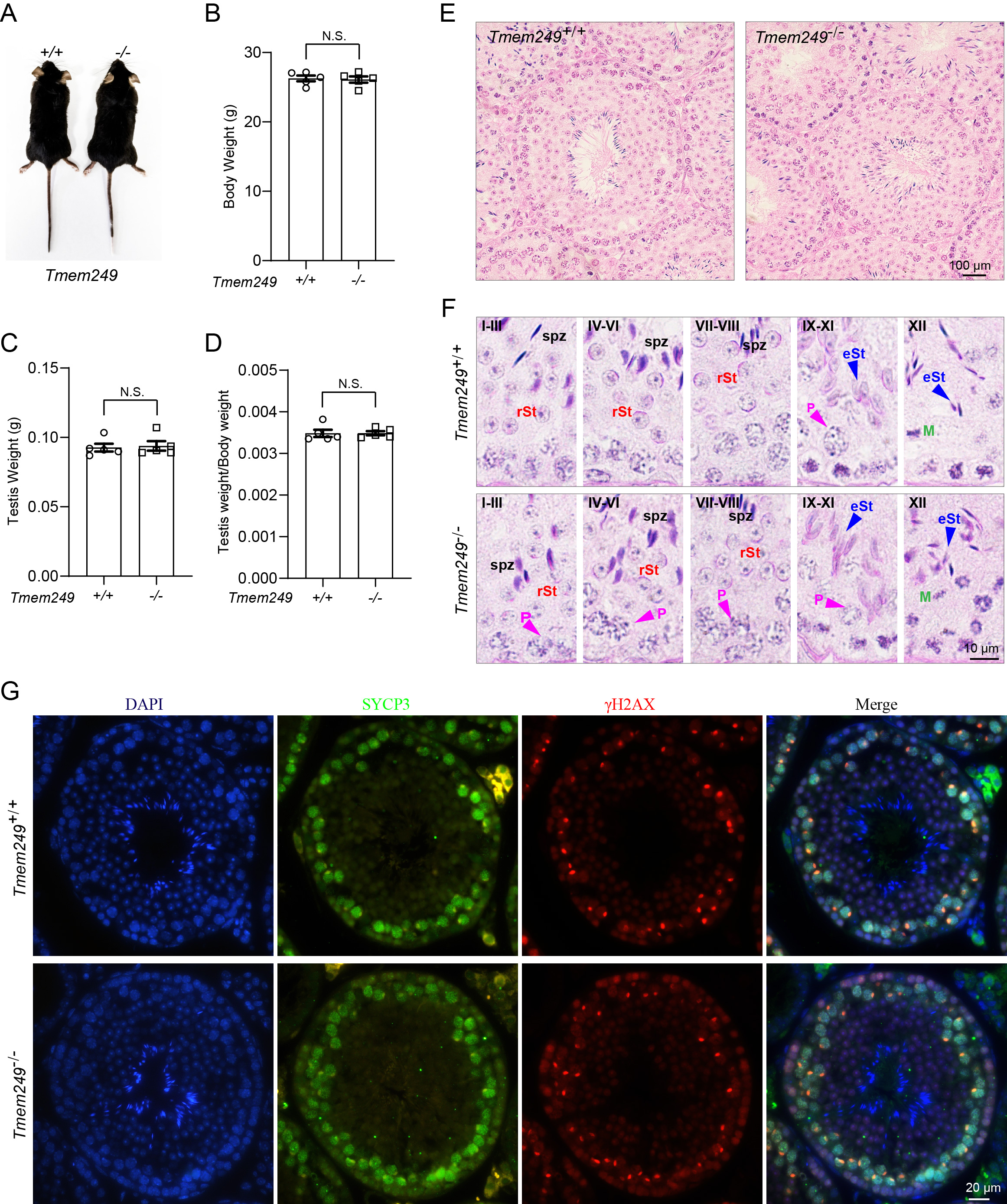


**Fig. S3.** The individual phenotype and testicular spermatogenesis of *Tmem249* mice. (A) Appearance characteristics of *Tmem249* mice. (B) Body weight of *Tmem249* mice. (C) Testis weight of *Tmem249* mice. (D) The ratio of testis weight to body weight from the *Tmem249* mice. (E) The structure of seminiferous tubules by the H&E staining of testis sections from the *Tmem249* mice. (F) PAS staining of different developmental stages of seminiferous tubules in *Tmem249* mice. The different stages of seminiferous tubules are indicated by roman numerals. P, pachynema; M, mitotic period; eSt, elongated spermatid; rSt, round spermatid; spz, spermatozoa.

**
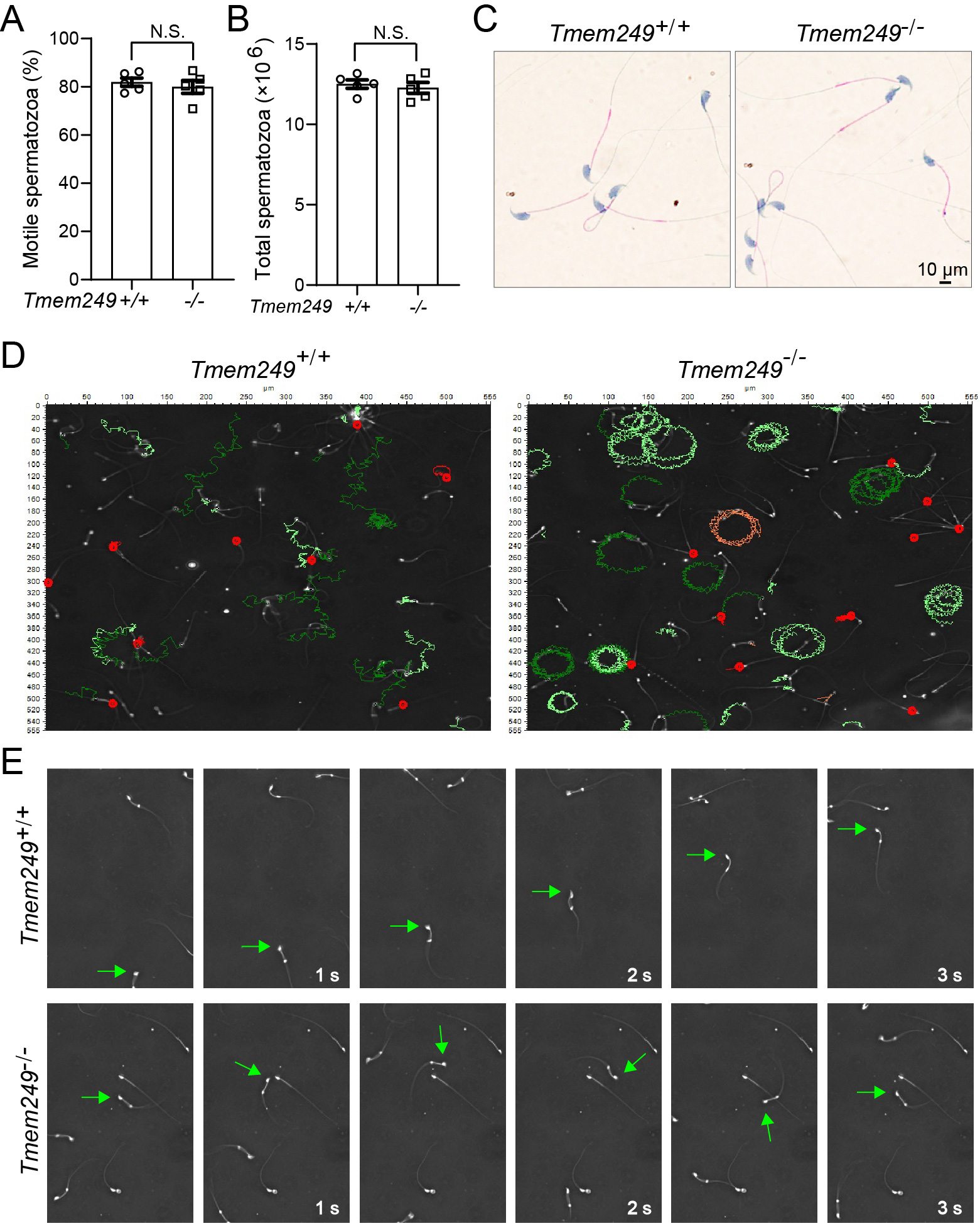
**

**Fig. S4.** Analysis of sperm quality of *Tmem249* mice. (A) Sperm motility from cauda epididymis of *Tmem249* mice. (B) Sperm counts from the unilateral cauda epididymis of *Tmem249* mice. (C) Papanicolaou (Pap) staining of sperm from cauda epididymis of *Tmem249* mice. (D) Sperm trajectories were tracked by the computer-assisted sperm analyzer. (E) Single sperm motion was recorded for 3 seconds by the computer-assisted sperm analyzer.
